# Improved connectivity and cognition due to cognitive stimulation in Alzheimer’s disease

**DOI:** 10.3389/fnagi.2023.1140975

**Published:** 2023-08-17

**Authors:** Qumars Behfar, Nils Richter, Merve Kural, Anne Clemens, Stefan Kambiz Behfar, Ann-Kristin Folkerts, Ronja Fassbender, Elke Kalbe, Gereon R. Fink, Oezguer A. Onur

**Affiliations:** ^1^Cognitive Neuroscience, Institute of Neuroscience and Medicine (INM-3), Juelich Research Centre, Jülich, Germany; ^2^Department of Neurology, Faculty of Medicine and University Hospital Cologne, University of Cologne, Cologne, Germany; ^3^Department of Information Systems, Geneva School of Business Administration (HES-SO Genéve), Carouge, Switzerland; ^4^Medical Psychology Neuropsychology and Gender Studies and Center for Neuropsychological Diagnostics and Intervention (CeNDI), Faculty of Medicine and University Hospital Cologne, University of Cologne, Cologne, Germany

**Keywords:** cognitive training, cognitive reserve, fMRI, compensation, plasticity

## Abstract

**Background:**

Due to the increasing prevalence of Alzheimer’s disease (AD) and the limited efficacy of pharmacological treatment, the interest in non-pharmacological interventions, e.g., cognitive stimulation therapy (CST), to improve cognitive dysfunction and the quality of life of AD patients are on a steady rise.

**Objectives:**

Here, we examined the efficacy of a CST program specifically conceptualized for AD dementia patients and the neural mechanisms underlying cognitive or behavioral benefits of CST.

**Methods:**

Using neuropsychological tests and MRI-based measurements of functional connectivity, we examined the (neuro-) psychological status and network changes at two time points: pre vs. post-stimulation (8 to 12 weeks) in the intervention group (*n* = *15*) who received the CST versus a no-intervention control group (*n* = *15*).

**Results:**

After CST, we observed significant improvement in the Mini-Mental State Examination (MMSE), the Alzheimer’s Disease Assessment Scale, cognitive subsection (ADAS-cog), and the behavioral and psychological symptoms of dementia (BPSD) scores. These cognitive improvements were associated with an up-regulated functional connectivity between the left posterior hippocampus and the trunk of the left postcentral gyrus.

**Conclusion:**

Our data indicate that CST seems to induce short-term global cognition and behavior improvements in mild to moderate AD dementia and enhances resting-state functional connectivity in learning- and memory-associated brain regions. These convergent results prove that even in mild to moderate dementia AD, neuroplasticity can be harnessed to alleviate cognitive impairment with CST.

## 1. Introduction

Alzheimer’s disease (AD) is a progressive neurodegenerative disease and the most common cause of dementia. AD patients show gradual but progressive cognitive impairments, and the overwhelming majority (80–90%) of AD patients with cognitive impairment will experience behavioral and psychological symptoms of dementia (BPSD) at some stage of the disease progression (Lyketsos et al., 2000, 2002). Prolonged hospitalization, increased health care costs and mortality are serious consequences of BPSD (Brodaty et al., 2015; Peters et al., 2015; Laganà et al., 2022), which result in existential suffering for patients and their families and a great burden on society.

In the absence of any cure for AD, the main medications used as symptomatic therapies include cholinesterase inhibitors and excitatory amino acid receptor antagonists with limited efficacy on the cognitive-mnestic symptoms of AD (Ballard et al., 2005; Sink et al., 2005; Campbell et al., 2008; Massoud and Léger, 2011; Sato et al., 2011). Recently approved anti-amyloid agents like Aducanumab and Lecanemab were only able to show a limited clinical effect, although changes of amyloid-imaging findings were strong and convincing (Budd Haeberlein et al., 2022; van Dyck et al., 2023). Further investigations and long-term follow-up are needed (van Dyck et al., 2023). Moreover, additional administrative and economic burden render difficulties for implementation of the current anti-amyloid agents in clinic. Given these limitations in pharmacological options, examining the effects of non-pharmacological interventions remains to be of interest.

A non-pharmacological approach intends to combat cognitive decline and could potentially reduce the severity of BPSD symptoms with comparable effectiveness to pharmacological treatment (Brodaty and Arasaratnam, 2012). Several non-pharmacological interventions have been used in managing cognitive impairment and BPSD in AD patients, including reminiscence, physical therapy, non-invasive brain stimulation, cognitive training, and cognitive stimulation (Abraha et al., 2017; Berg-Weger and Stewart, 2017). To date, the vast majority of dementia management guidelines still recommend non-pharmacological interventions including cognitive stimulation therapy (CST) as a first line of treatment (Li et al., 2022).

Cognitive stimulation therapy as one of the most popular non-pharmacological approaches comprises various group activities and discussions to enhance the general cognitive and social functioning (Woods et al., 2012, 2023). Systemic reviews and meta-analyses have provided evidence for significant cognitive improvement induced by CST in mild to moderate AD dementia stages (Buschert et al., 2010; Aguirre et al., 2013; Alves et al., 2013; Huntley et al., 2015; Chen, 2022; Woods et al., 2012, 2023). Moreover, for a relatively smaller number of studies, a clinically promoting effect of CST for several outcome measures including quality of life (QoL) has been reported (Buschert et al., 2010; Aguirre et al., 2013; Woods et al., 2012, 2023).

The initial research evaluating the effects of CST in AD lacked data on its neurobiological mechanisms (Buschert et al., 2010). Language has been suggested as the most likely modifiable domain owing to the structure and nature of activities in CST (Spector et al., 2010; Lobbia et al., 2019), However, the literature is fragmented and inconclusive as to the mechanisms. More recent evidence and theoretical frameworks indicate that CST-modulated improvement of cognition in people with dementia may relate to brain reserve and cognitive reserve (Liu et al., 2017). Nevertheless, the underlying mechanisms are still not fully understood. Traditional cognitive approaches assume that compensatory cognitive strategies, which are thought to be more common in people with higher education and a cognitively demanding daily life, are the reason for maintaining cognitive performance levels despite brain damage. Therefore, in earlier and more recent studies, years of formal education have been widely suggested as the most robust component of cognitive reserve (Roe et al., 2007; Jefferson et al., 2011; Farfel et al., 2013; Wilson et al., 2019).

Neuroimaging studies show that with better functional connectivity and higher efficiency in different cortical regions, cognitive performance levels can be maintained longer (Vuoksimaa et al., 2013). Moreover, it has been proposed that CST can alter neuronal excitability, promoting brain plasticity and compensatory mechanisms (Cespón et al., 2018). Despite present evidence and potential for its further cognitive benefits, the precise mechanism of CST remains unclear.

Due to the complicated nature of CST, unraveling its mechanisms without knowledge of brain-level activities has proven challenging. Brain connectivity analysis offers an opportunity to advance our knowledge of the underlying processes of non-pharmacological interventions such as CST. Nevertheless, the growing body of research assessing the effects of CST in AD dementia contrasts with the shortage of imaging studies, despite the fact that imaging studies typically reveal higher effects compared to pure cognitive investigations (Rose and Donohoe, 2013). Functional magnetic resonance imaging (fMRI)-based brain network connectivity analysis may provide experimental data on putative neuropsychological underlying mechanisms of CST. Brain network connectivity can change as a result of behaviorally relevant experience, which is referred to as activity-dependent plasticity (Ganguly and Poo, 2013). As a result of learning, topologically complex or globally dispersed brain networks may also undergo remodeling (Bassett et al., 2011). Therefore, combining CST with fMRI might provide innovative comprehension of how fundamental brain plasticity mechanisms operate in CST. In addition, studies with a follow-up assessment, which examine the extent and duration of potential CST effects, are warranted.

This study assessed (i) the efficacy of a CST program, composed of sixteen 60-min sessions delivered twice weekly over 8 weeks, with follow-up assessment after 3 months, and (ii) the underlying neural correlates using magnetic resonance imaging (MRI).

We hypothesized that CST induces positive effects on global cognition, BPSD, and QoL in mild to moderate AD dementia. We also explored whether cognitive improvements after CST are associated with an enhancement in brain connectivity which support memory and cognition. Herein, we related the pre- vs. post-stimulation imaging findings and the neuropsychological outcomes of the intervention compared to the no-intervention control group.

## 2. Materials and methods

### 2.1. Study design

**FIGURE 1:**
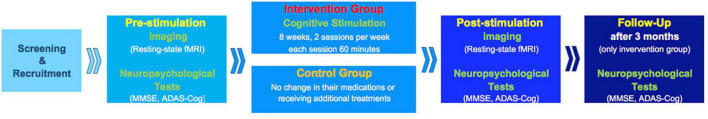
Study flow. Schematic illustration of the study flow.

Initially, the eligibility of potential participants was assessed via phone. The subjects were then invited over to the clinic to be closely checked on their participation eligibility according to the inclusion and exclusion criteria. The aim and processes of the study were explained to the participants and their relatives. Following written consent, the baseline neuropsychological assessment (pre-stimulation) was performed. This was followed by 8 weeks of CST for the intervention group, while the control group did not have an appointment during this period. Under the criteria recommended by Bruer et al. (2007), all pre- and post-stimulation assessments, including neuropsychological tests and neuroimaging, were conducted within 1 week before the beginning of the stimulation period or after the last session of the stimulation program (see Figure 1). A neuropsychological follow-up was performed 3 months after the post-stimulation assessment.

### 2.2. Participants

The Ethics Committee of the University of Cologne, Germany, approved this study (ID:16-298), and the research was conducted following the Declaration of Helsinki 1975. Each patient or legal or authorized representative gave informed written consent before the study began. At the end of the study, control group participants were offered to participate in the intervention program.

Participants and their caregivers were interviewed to obtain information on their demographics, including precise years of education as a proxy for cognitive reserve. Individuals with mild to moderate AD dementia were recruited from the Neurology department of the University Hospital of Cologne, Germany. The study is composed of an intervention and a control group, and all recruited participants in both groups fulfilled the criteria for Alzheimer’s continuum according to Jack et al. (2018) (see Table 1) with at least CSF or PET-based amyloid positivity. Attribution of dementia severity was determined by qualified neurologists (OAO and NR) at the neurology department of Cologne university hospital, and operationalized by a Mini-Mental State Examination (MMSE) score between 10 and 26 points (Folstein et al., 1975).

**TABLE 1:** CSF and imaging biomarkers of AD.

Aβ42 (pg/ml)	pTAU(pg/ml)	tTAU(pg/ml)	Aβ42/40	tTAU/Aβ42	FDG-PET (metaROI)	18F-AV-45 (SUVR)	AT(N)- profile
***Intervention group***							
869.5	121	506.7	0.07	0.58	N/A	N/A	A+T+(N)+
399.5	62	293.1	0.08	0.73	N/A	N/A	A+T+(N)-
516.6	123	522.1	0.06	1.01	N/A	N/A	A+T+(N)+
205.1	80	287.7	0.05	1.40	N/A	N/A	A+T+(N)-
372.9	72	464.3	0.05	1.25	N/A	N/A	A+T+(N)-
408.8	38	210.4	0.08	0.51	N/A	N/A	A+T-(N)-
491	82	202	0.07	0.41	N/A	N/A	A+T+(N)-
680.3	100	687.2	0.06	1.01	N/A	N/A	A+T+(N)+
313.9	112	911.1	0.05	2.90	N/A	N/A	A+T+(N)+
721.3	119	1662.8	0.05	2.31	N/A	N/A	A+T+(N)+
529	78	660.8	0.05	1.25	N/A	N/A	A+T+(N)+
427	123	811	N/A	1.90	N/A	N/A	A+T+(N)+
715.3	174	511.4	0.05	0.71	N/A	N/A	A+T+(N)+
368.7	92	424.1	0.05	1.14	N/A	N/A	A+T+(N)-
752.9	121	722.2	0.06	0.99	N/A	N/A	A+T+(N)+
***Control group (self recruitment)***							
361.5	67	292	0.05	0.81	N/A	N/A	A+T+(N)-
545.6	116	1164.6	0.04	2.13	N/A	N/A	A+T+(N)+
527 474.6 517.6 340 566.3 426.2	N/A 71 83 48 67 99	335 293.2 562.1 365 219.1 409.6	0.06 0.07 0.06 N/A 0.07 0.08	0.64 0.62 1.09 1.07 0.38 0.88	N/A N/A N/A N/A N/A N/A	N/A N/A N/A N/A N/A N/A	A+T?(N)+ A+T+(N)- A+T+(N)+ A+T-(N)- A+T+(N)- A+T+(N)-
***Control group (ADNI)***							
564.5	21.54	224.6	N/A	0.4	1.03	1.45	A+T-(N)+
523.9	24.81	266.2	N/A	0.51	1.05	1.67	A+T+(N)+
801.1	17.29	211.7	N/A	0.26	0.92	N/A	A+T-(N)+
805.3	24.39	268.1	N/A	0.33	0.91	1.23	A+T+(N)+
624.1	37.72	365.8	N/A	0.59	1.02	1.45	A+T+(N)+
760	63.4	606.6	N/A	0.8	1.21	1.60	A+T+(N)+
461.2	23	247.1	N/A	0.05	1.22	1.39	A+T+(N)+

Biomarkers suggestive of AD were gleaned for all subjects. Norms for the intervention group and the control (local-recruitment) are: Aβ42 > 650 pg/ml, pTAU < 61 pg/ml, tTAU < 466 pg/ml; Aβ42/40 ratio > 0.1; tTAU/Aβ42 < 0.52. Norms for the control group (ADNI) are: Aβ42 > 880 pg/ml; pTAU < 23 pg/ml; tTAU < 93 pg/ml; tTAU/Aβ42 < 0.33; FDG-PET > 1.21 metaROI; ^18^F-AV-45 < 1.11 SUVR. Aβ42, amyloid-beta 42; pTAU, phosphorylated tau-Protein; tTAU, Total Tau-Protein; FDG-PET, ^18^F-fluorodeoxyglucose-positron emission tomography; ^18^F-AV-45, Florbetapir F 18 for amyloid-beta plaques positron emission tomography imaging.

Other recruitment criteria included age older than 60 years, normal or corrected-to-normal vision and hearing, and being a native German speaker or having excellent proficiency in the German language. Patients with life-threatening conditions or other concomitant neurological or psychiatric disorders were excluded. To avoid any selection bias on the one hand side and to start the intervention in a timely manner after patient consent for the study, the patients fulfilling the inclusion criteria were recruited consecutively for the intervention group, which was directly followed by the recruitment for the control group in the same manner.

Participants did not receive remuneration. All MRI scans were obtained at the Research Centre Juelich, and for all participant either a transport service to/from the Research Centre Juelich was arranged or they received travel cost reimbursement.

#### 2.2.1. Intervention group

Participants of the intervention group visited the multi-domain CST program *NEUROvitalis senseful*, specifically developed for AD dementia patients, based on the previously published standardized cognitive training program *NEUROvitalis* (Baller et al., 2009). Liesk et al. (2015) previously concluded that the original *NEUROvitalis* cognitive training program designed for healthy elderly and patients with MCI included parts, e.g., psychoeducational elements and training of cognitive strategies, that are too challenging for AD dementia patients. Accordingly, these demanding parts were removed for *NEUROvitalis senseful* while the group games remained in the modified version. In addition, the CST program covers a broader range of cognitive domains to ensure comprehensive stimulation. Besides, tasks for sensory stimulation and relaxation were embedded. Each session follows a standardized framework, which is briefly outlined in Table 2. The standard group CST protocol calls for 14 sessions to be administered twice weekly for 7 weeks, with a focus on social and information processing (Spector et al., 2003), proposing modest efficacy similar to pharmacological treatments (Spector et al., 2003; Onder et al., 2005). On the premise to provide the required “dose” of stimulation to combat cognitive decline, *NEUROvitalis senseful* was built in accordance with the experience obtained from the previously most established CST programs with a median session length of 45 min, a median frequency of twice a week, a median total number of 20 sessions, and a median follow-up length of 10 weeks (Woods et al., 2023). Certified neuropsychologists conducted all stimulating interventions with a maximum of four participants per groups, a frequency of two sessions per week, and an overall duration of 8 weeks. Each of the 16 conducted sessions contained various group activities and lasted for 60 min. Every session begins with a brief ritual called the “mood scale” where each person can share his or her mood. An exercise focusing on one of the four cognitive domains–executive functions, memory, language, or social cognition–is included in the first main phase, which is subsequently followed by a short relaxation exercise from one of three domains. The activities in this section were inspired by the well-established techniques of progressive muscle relaxation (Jacobson et al., 1990) and mindfulness (Bishop et al., 2004). The second phase consists of sensory-stimulating activities such as brief narratives with light movement tasks or tactile, olfactory, or auditory stimulation. Cognitive and sensory exercises were designed to establish a personal connection to the biographies of the group members and elicit discussions about their experiences (Werheid and Thöne-Otto, 2006). The majority of exercises can be customized to the individual capacities of people with dementia. For example, providing more or fewer cues or options for selection allows the chance to lower or enhance the level of difficulty in the memory, word finding, and sensory stimulation sections. The activities in each phase are evenly distributed across the 16 intervention sessions, ensuring that all domains are equally stimulated during intervention period. As a whole, our CST program focuses on stimulating cognitive functions, while incorporating additional elements to promote cognition. As a result, sensory stimulation tasks, requiring comprehensive recognition and verbalization of haptic and olfactory information, stimulate cognitive functions. Likewise, movement tasks target procedural memory. Each session includes a relaxing period, which may strength memory, mental receptiveness, and concentration (Schloffer et al., 2010). The content of the stimulating interventions was described in detail by Middelstadt et al. (2016).

**TABLE 2:** Structure, sessions and phases of the cognitive stimulation program NEUROvitalis senseful.

Duration	5 min	20 min					10 min				20 min					5 min
Session number	Mood scale	Exercises for cognitive functions	Executive functions	Memory	Language	Social cognition	Relaxation period	Progressive muscle relaxation	Slight movement	Mindfulness	Sensory stimulation	Tactile	Olfactory	Auditory	Body language	Mood scale
1	✓	Everyday situations				✓	Hands	✓			Describe fragrances		✓			✓
2	✓	Think differently	✓				Pass it forward!		✓		Describe fragrances		✓			✓
3	✓	Think differently	✓				Seeing			✓	Movement story				✓	✓
4	✓	Meaningful pictures		✓			Arms, shoulders, neck	✓			Describe sounds			✓		✓
5	✓	Finding words			✓		Finger gymnastics		✓		Describe sounds			✓		✓
6	✓	Everyday situations				✓	Tasting			✓	Feeling exercises	✓				✓
7	✓	City map game	✓				Hands	✓			Feeling exercises	✓				✓
8	✓	Finding words			✓		Cups and balls		✓		Movement story				✓	✓
9	✓	Finding words			✓		Seeing and feeling			✓	Movement story				✓	✓
10	✓	Meaningful pictures		✓			Stomach, back, buttock	✓			Describe fragrances		✓			✓
11	✓	Basic emotions				✓	Finger gymnastics		✓		Describe sounds			✓		✓
12	✓	Basic emotions				✓	Seeing and feeling			✓	Feeling exercises	✓				✓
13	✓	Finding words			✓		Legs and feet	✓			Movement story				✓	✓
14	✓	Category memory game		✓			Pass it forward!		✓		Describe fragrances		✓			✓
15	✓	Category memory game		✓			Tasting			✓	Describe sounds			✓		✓
16	✓	City map game	✓				Hands	✓			Feeling exercises	✓				✓

Importantly, participants were also asked to avoid any change in their medication regimen, participation in any other interventional studies or commencing any new therapy. And, at the end of the CST, the participants’ compliance to these instructions were confirmed with the participants and their family members.

#### 2.2.2. Control group

Controls did not receive any intervention and were asked to maintain their usual daily routines and avoid parallel medical or any other therapeutic interventions. By the second scan, the commitment of the participants to these requirements were confirmed by the participants and their family members. Due to the COVID-19 pandemic and the following measures to reduce contacts from 2020 onward, we could not continue recruiting patients for the control group. Thus, to meet the requirement that the control and intervention groups comprise an equal number of participants, we decided to use the Alzheimer’s Disease Neuroimaging Initiative (ADNI) database (adni.loni.usc.edu). The AD patients from the ADNI database (*n* = 7), with at least two imaging sessions and neuropsychological evaluations, were meticulously selected to match with our recruitment and study design criteria and to maintain insignificant difference between the intervention and the control group, considering demographic and diagnostic measures, as well as MRI scanner type, temporal distance between two imaging sessions and any change in the concurrent medication (see Table 3). However, only MMSE and ADAS-Cog scores were available for the ADNI subjects. The cut-off points for each of the considered biomarkers from the ADNI database were derived from international labs (Jagust et al., 2009; Landau et al., 2012; Hansson et al., 2018; Ou et al., 2019), and each participant’s AT(N)-profile was determined according to Jack et al. (2018) (see Table 1).

**TABLE 3:** Demographic data.

	Intervention group	Control group	Control group (self)	Control group (ADNI)	Intervention vs. Control *p*-value
No. of Pat.	15	15	8	7	_
Sex (male %)	60.00%	60.00%	62.50%	57.00%	1
Age	72.5 ± 2.2	73.7 ± 1.6	72 ± 0.5	75.6 ± 1.3	0.8
MMSE	19.6 ± 1	22.8 ± 1	21.2 ± 1.6	24.5 ± 0.6	0.16
Education (years)	13.6 ± 0.7	14.7 ± 0.8	13.3 ± 0.8	16.2 ± 1.4	0.6

ADNI, Alzheimer’s Disease Neuroimaging Initiative; MMSE, Mini-Mental State Examination.

### 2.3. Neuropsychological assessments

Identical neuropsychological tests were performed in pre- and post-stimulation assessment for each intervention and control group participant. All neuropsychological tests are used in international research studies (Moniz-Cook et al., 2008). The MMSE (Folstein et al., 1975) and the Alzheimer’s Disease Assessment Scale, cognitive subsection (ADAS-Cog) (Rosen et al., 1984), were used to evaluate the participants’ cognitive performance by providing a quantitative assessment of cognitive functions. These well-established measures have been successfully employed in earlier cognitive stimulation studies (Spector et al., 2003; Orrell et al., 2014; Werheid et al., 2021), and potentiated the comparability and replicability of the results. In addition, these measurements comprise multiple domains, provide a broader view on cognitive and memory functions, and are less susceptible to false-positives. The MMSE score typically ranges from 0 to 30, with a higher score indicating better cognitive performance. There are several cutoff points and ranges to spot the presence of cognitive impairment and to classify the level impairment (Tombaugh and McIntyre, 1992; Folstein et al., 2001; Ruchinskas and Curyto, 2003), nevertheless, reporting the specific score allows for clear and easy comparison across individuals and tracking changes over time. The ADAS-Cog is widely used in clinical trials with AD patients (Hobart et al., 2013) and is composed of 11 items to measure memory, orientation/praxis, and language. The ADAS-Cog score spans 0 to 74 points, with greater scores indicating more significant cognitive impairment. Besides, subjective and objective assessments of QoL were conducted through the European Quality of Life Five Dimension with Five Levels (EQ-5D-5L) questionnaire, a widely used multi-attribute utility tool to measure health-related quality of life (EuroQol Research Foundation, 2019). It consists of five items, including mobility, self-care, usual activities, pain (or discomfort), and anxiety (or depression), to measure various aspects of life. The total sum score ranges between 0 and 100 points. A greater sum score indicates a greater level of QoL.

Further assessments were mainly BPSD oriented and included the Neuropsychiatric Inventory (NPI) (Cummings, 1997), as well as the Alzheimer’s Disease Cooperative Study-Activities of Daily Living Inventory (ADCS-ADL) (Galasko et al., 2006). The NPI is composed of twelve items to evaluate various neuropsychiatric symptoms, including anxiety, apathy/indifference, agitation/aggression, aberrant motor behavior, appetite/eating disturbances, delusions, depression/dysphoria, disinhibition, euphoria/elation, hallucination, irritability/lability, and nocturnal behavioral disturbances. For each domain, a score is generated by the multiplication of the frequency and severity of the enquired symptoms, yielding a composite score ranging from 0 (no behavioral symptom) to 144 points (maximum severity of all symptoms).

The ADCS-ADL assesses the activities of daily living (ADL) over the previous 4 weeks. The total score ranges between 0 (lowest functional ability) and 78 (highest functional ability) points, the sum of all items.

The neuropsychological assessments were administered as a structured interview. EQ-5F-5L and NPI assessments contained, along with a self-rated, an additional proxy-rated section for which each patient’s spouse was interviewed.

In the control group, seven subjects were selected form the ADNI database, matched with our recruitment and study design. Notably, as for the cognitive and neuropsychological tests, only MMSE and ADAS-Cog scores were available for the ADNI subjects.

### 2.4. MRI data acquisition and preprocessing

Participants in the study with no contraindication for MRI were scanned at the Research Centre Juelich. Structural MRI and resting-state functional MRI were obtained at a 3T MAGNETOM Trio scanner (Siemens, Erlangen, Germany). T1 structural images were acquired using a rapid gradient-echo sequence with the following parameters: repetition time (TR) = 2,250 ms, echo time (TE) = 3.03 ms, flip angle (FA) = 9°, field of view (FOV) = 256 mm × 256 mm, voxel size = 1 mm isotropic, 176 gapless interleaved sagittal slices. During the 7-min resting-stage image acquisition, patients were instructed to stay awake, look at a projected cross sign, and not think of anything particular. For the functional images, echo-planar imaging (EPI) with the following parameters was used: TR = 3,000 ms, TE = 30 ms, FA = 90°, FOV = 200 × 200, voxel size = 2.5 × 2.5 × 2.8, interleaved oblique slices parallel to the infra-supratentorial line with a gap of 0.28 mm. The seven subjects of the control group, whose data were taken from the ADNI database, had also been scanned on 3T Siemens MR scanners. T1 structural images were acquired using a rapid gradient-echo sequence with the following parameters: slice thickness = 1.0 mm and Matrix *Z* = 176.0. The following parameters were used for the functional images: echo planar imaging (EPI) with a TR = 3,000 ms, TE = 30 ms, and slice thickness of 2.5∼3.4 mm.

Magnetic resonance images were preprocessed using the default preprocessing pipeline of the CONN toolbox (Whitfield-Gabrieli and Nieto-Castanon, 2012). The first four images of 155 volumes were removed to allow the signal to reach equilibrium. Functional images were realigned to the first acquired volume in the session. Next, echo-planar images (EPIs) were co-registered to the high-resolution T1 structural image, normalized to the Montreal Neurological Institute (MNI) stereotactic space, and resampled at 2 mm× 2 mm× 2 mm voxel size. After normalization, images were spatially smoothed with an 8-mm full-width at half maximum (FWHM) isotropic Gaussian kernel. Head motion parameters were individually controlled and excluded at a ± 3 mm relative displacement criterion.

To consider motion-related artifacts (Conwell et al., 2018), we incorporated frame-wise displacement (FD), calculated according to Jenkinson et al. (2002), employed in our models as a covariate of no interest. The proposed method by Jenkinson et al. (2002) is preferred over other FD methods as it accounts for voxel-wise differences in its derivation (Yan et al., 2013).

### 2.5. Brainnetome Atlas

The extended version of the Brainnetome Atlas, also covering the cerebellum, includes 274 ROIs (210 cortical and 36 subcortical and 28 cerebellar subregions), which are assigned to brain functions based on numerous meta-analyses of tasked-based functional imaging studies (Fan et al., 2016). Most of the available brain atlases lack fine-grained parcellation and fail to provide all functional aspects of the brain regions. Using various multimodal imaging techniques, the Brainnetome Atlas was developed to provide a connectivity-based parcellation framework, which determines the subsections of the human brain, revealing new dimensions of connectivity architecture. Specifically, the Brainnetome Atlas merges brain connectivity with microscale information such as cytoarchitecture of various brain regions. The structures in the Brainnetome Atlas are related to mental processes via the BrainMap database (Laird et al., 2009; Balsters et al., 2014; Fox et al., 2014), thereby providing an evaluation of the mental processes maintained by each cortical and subcortical region of the Brainnetome Atlas (Fan et al., 2016). The functionalities of each subarea in the Brainnetome Atlas are characterized, based on the behavioral domains and paradigm class metadata labels of the BrainMap database,^1^ using forward and reverse inferences (Eickhoff et al., 2011; Cieslik et al., 2013; Clos et al., 2013; Fox et al., 2014; Fan et al., 2016).

### 2.6. Structural analysis

Structural images were analyzed using the voxel-based morphometry (VBM) technique on CAT12 toolbox and SPM12 software, which provide thorough details on brain morphometric characteristics while averting biases brought on by structural variations (Ashburner and Friston, 2000). T1 structural images were first segmented and smoothed using CAT12 toolbox. Total brain volume was calculated as the sum of grey matter (GM), white matter (WM); and the total intracranial volume (TIV) as the sum of GM, WM, and CSF volumes. Then, paired-sample *t*-test and ANCOVA were conducted using SPM12 toolbox with TIV as a nuisance covariate to explore the within and between group comparisons.

### 2.7. Functional connectivity analyses

Two different approaches were used: (1) atlas-based ROI-to-ROI functional connectivity analysis, and (2) seed-to-voxel analysis using *a priori* selection derived from the ROI-to-ROI results. For the atlas-based ROI-to-ROI method, we incorporated the Brainnetome Atlas (Fan et al., 2016) with 274 ROIs on the CONN toolbox v.19.c (Whitfield-Gabrieli and Nieto-Castanon, 2012) to generate connectivity matrices for each subject, averaging the time series of the BOLD (blood oxygenation level dependent) signals of all voxels in each ROI of the Brainnetome Atlas and calculating and z-transforming the Pearson’s correlation of these average signals between ROIs. Likewise, in the seed-to-voxel analysis, the correlation maps on the whole brain were produced by extracting the BOLD signal from the seed ROI, computing and z-transforming the correlation coefficient between that signal and the signals from all other brain voxels.

Following ROI-to-ROI and voxel-based analyses at the subject level, general linear models were fitted using all corresponding within-subject pairwise z-transformed correlation coefficient measures. Then, the group-level ROI-to-ROI analysis (*p* < 0.05, *p*-FDR seed-level corrected) and seed-to-voxel analysis at a voxel-wise threshold of *p* < 0.001 and a cluster-level threshold of *p* < 0.05 (FDR-corrected) were performed. FD was included as a covariate of no interest for all within and between group contrasts. After group-level comparisons, for each subject in the ROI-to-ROI analysis, the z-transformed correlation coefficients, and in the seed-to-voxel analysis the fisher z-transformed correlation coefficients averaged over all voxels of the cluster, were extracted for further correlation analysis with the neuropsychological tests.

### 2.8. Association between connectivity changes and neuropsychological tests

We examined the correlation of the connectivity changes in ROI-to-ROI and Seed-to-Voxel analyses with the cognitive measures among the participants in the intervention groups after the CST. To do so, for each subject in the ROI-to-ROI analysis, the fisher z-transformed correlation coefficients and, in the seed-to-voxel analysis, the fisher z-transformed correlation coefficients averaged over all voxels of the cluster were extracted from the resting-state MR images as the connectivity measures, and their association with the changes of MMSE and ADAS-Cog scores after the CST were estimated using linear regression models with adjustment for age and sex.

### 2.9. Statistical analyses

Statistical analyses were performed using IBM SPSS, version 23.0, MATLAB R2017b (The MathWorks, Natick, MA, USA), and R (R Core Team, 2013). Before the application of the statistical analyses on the data, reliability of the changes in MMSE, ADAS-Cog, and EQ-5D-5L measures was controlled through the reliable change index (RCI) (post- to pre-stimulation and follow-up to post-stimulation) in the intervention group and (2nd test to 1st test) in the control group. Besides, the normal distribution of the assessments’ data was confirmed using the Shapiro–Wilk test for each group. A *post hoc* estimation of our sample size using G*Power 3.1 and IBM SPSS was also performed.

Based on our hypotheses, we analyzed the effect of stimulation in the intervention group versus any changes in the control group in two levels. In the first step, within-group changes were evaluated using multifactorial ANOVA in each group. To eliminate the batch effect in the statistical models, a covariate indicating whether the participant was initially recruited for the study or the data was imported from ADNI was taken into consideration. Next, to assess the between-group changes, we juxtaposed the within-group changes in the intervention group (post–stimulation > pre-stimulation) against the within-group changes in the control group (2nd > 1st). Due to the relatively small sample size, the between group comparison was performed using Wilcoxon test as the non-parametric test of choice. For all within- and between-group contrasts, the significance level was set at α = 0.05.

## 3. Results

The imaging and the neuropsychological data of all participants in the intervention and the control groups were assessed. In the intervention group, with a 17% (*n* = 3) drop-out ratio, 83% (*n* = 15) of participants successfully completed the program, among whom 72% (*n* = 13) underwent MR imaging. In the locally recruited subgroup of the controls (*n* = 8), 75% (*n* = 6) of the participants were compatible with our MR safety criteria and received MR scans. For the obtained data from ADNI, (*n* = 7), only imaging data, MMSE, and ADAS-Cog scores were available. The outcome of psychological interventions such as CST have been shown to be affected by individual characteristics particularly age and education (Carbone et al., 2021). Therefore, we ascertained the insignificant difference between the intervention and the control groups on the variables including the baseline MMSE, age and education, the latter serving as a proxy for cognitive reserve (see Table 3).

### 3.1. Neuropsychological assessments

**FIGURE 2:**
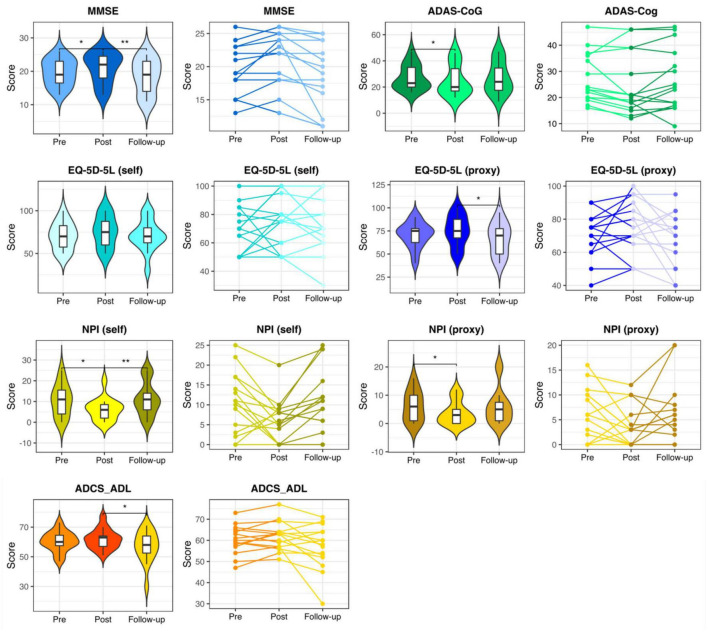
Neuropsychological assessments in the intervention group. Pre-stimulation, post-stimulation, and follow-up results of the neuropsychological assessments in the participants of the intervention group. MMSE, Mini-Mental State Examination; ADAS-Cog, the Alzheimer’s Disease Assessment Scale, cognitive subsection; EQ-5D-5L, the European Quality of Life Five Dimension with Five Levels; NPI, The Neuropsychiatric Inventory; ADCS-ADL, the Alzheimer’s Disease Cooperative Study-Activities of Daily Living Inventory. * and ** respectively represent *p* < 0.05 and *p* < 0.01.

Mini-Mental State Examination, ADAS-Cog, NPI (self), and NPI (proxy) measures showed significant changes in the pre- vs. post-stimulation contrast in the intervention group, indicating that the participants improved from baseline. Furthermore, at follow-up, a significant return to their baseline values was observed. While the self- and proxy-rated EQ-5D-5L along with ADCS-ADL revealed no significant improvement after the stimulation period, at follow-up, the proxy-rated EQ-5D-5L and ADCS-ADL score showed a significant worsening compared to the post-stimulation assessment (see Figure 2 and Supplementary Table 1).

**FIGURE 3:**
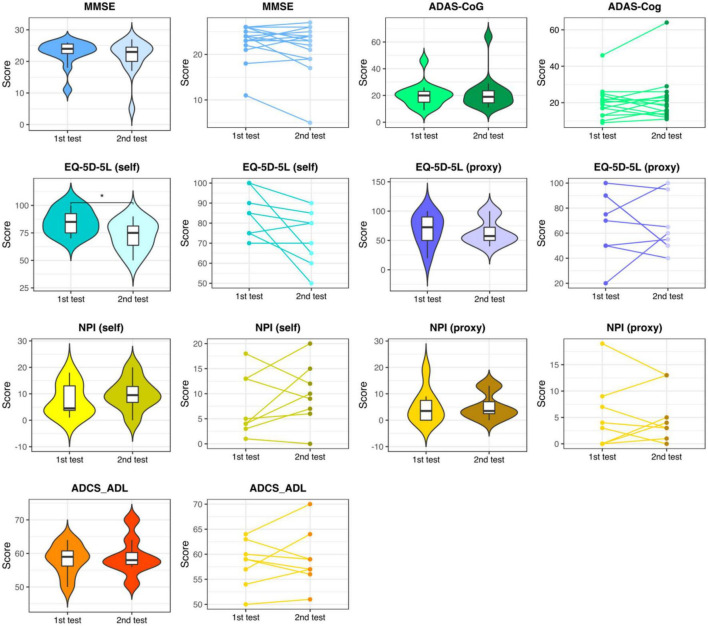
Neuropsychological assessments in the control group. Results of the 1^st^ and the 2^nd^ neuropsychological assessments with a distance of approx. 10 weeks in the control group. MMSE, Mini-Mental State Examination; ADAS-Cog, the Alzheimer’s Disease Assessment Scale, cognitive subsection; EQ-5D-5L, the European Quality of Life Five Dimension with Five Levels; NPI, The Neuropsychiatric Inventory; ADCS-ADL, the Alzheimer’s Disease Cooperative Study-Activities of Daily Living Inventory. * respectively represent *p* < 0.05.

In the control group, except for the self-rated EQ-5D-5L, which showed a significant worsening of QoL, no significant change was observed (see Figure 3 and Supplementary Table 1).

Juxtaposing the within-group changes in the intervention group (post-stimulation > pre-stimulation) against the within-group changes in the control group (2nd > 1st) revealed a significant difference in MMSE, EQ-5D-5L (self-rated) and NPI (self-rated) measures in favor of the intervention group (see Table 4).

**TABLE 4:** Outcomes of neuropsychological assessments.

Intervention vs. Control		
Assessment	Intervention_post–pre_ vs. Control_2nd_-_1st_	
	Wilcoxon test	
	Effect size	(*p*-value)
MMSE	0.38	**0.04**
ADAS-Cog	0.24	0.1
EQ-5D-5L (self)	0.44	**0.03**
EQ-5D-5L (proxy)	0.23	0.2
NPI (self)	0.45	**0.03**
NPI (proxy)	0.28	0.1
ADCS-ADL	0.03	0.9

MMSE, Mini-Mental State Examination; ADAS-Cog, the Alzheimer’s Disease Assessment Scale, cognitive subsection; EQ-5D-5L, the European Quality of Life Five Dimension with Five Levels; NPI, The Neuropsychiatric Inventory; ADCS-ADL, the Alzheimer’s Disease Cooperative Study-Activities of Daily Living Inventory. Statistically significant p-values are bolded.

**FIGURE 4:**
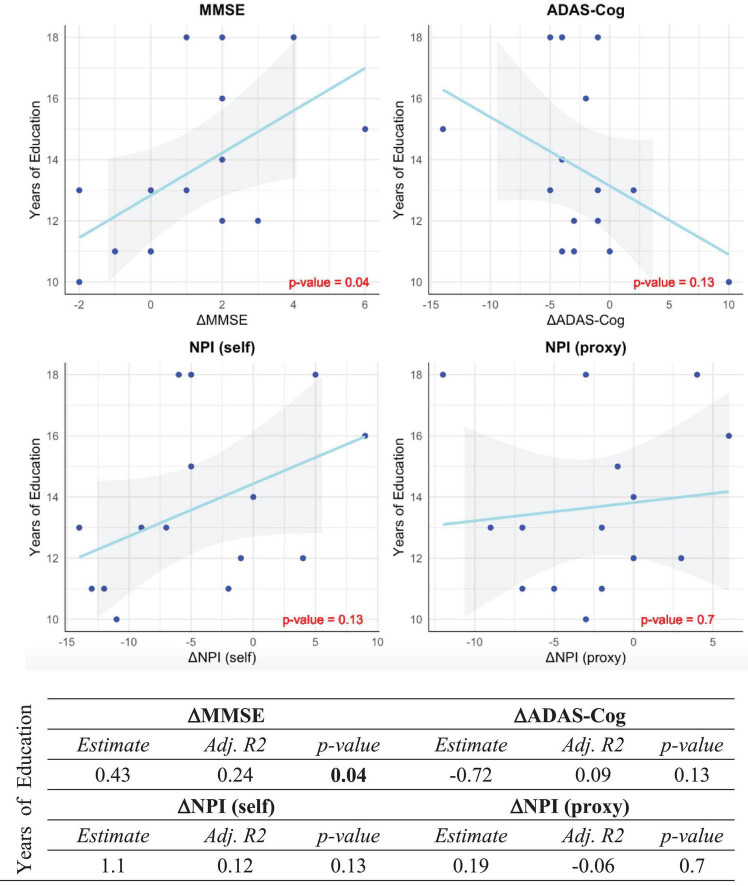
Association between years of education and significant outcomes of CST. Correlation between years of education as a proxy measure for cognitive reserve and post- vs. pre-stimulation changes of MMSE, ADAS-Cog, NPI (self) and NPI (proxy) scores in the intervention group. The post- vs. pre-stimulation changes are, respectively represented by ΔMMSE, ΔADAS-Cog, ΔNPI (self) and ΔNPI (proxy). All *p*-values are Bonferroni-corrected for multiple comparisons. Statistically significant *p*-values are bolded.

On the questioned premise of the relevance of cognitive reserve as a predictor of the intervention response in CST, we assessed the association of years of education as a proxy for cognitive reserve with the change of MMSE, ADAS-Cog, NPI (self), and NPI (proxy) measures which showed significant improvement after CST. As shown in Figure 4, among MMSE, ADAS-Cog, NPI (self), and NPI (proxy) which showed improvement after CST, MMSE was significantly associated with years of education, which indicates the predictability of cognitive gain after CST based on the cognitive reserve.

### 3.2. VBM analysis

In both groups, paired *t*-test did not show any significant structural changes over time and ANCOVA did not reveal any significant difference in the contrast between the two groups. Next, we speculated whether the base line total brain volume could predict the intervention response in CST. Herein, we assessed the association of the base line total brain volume as an indicator of brain reserve with the changes of MMSE, ADAS-Cog, NPI (self) and NPI (proxy) measures which showed significant improvement after CST. As shown in Supplementary Figure 1, improvement in MMSE, ADAS-Cog, NPI (self) and NPI (proxy) measure after CST in the intervention group did not show any significant association with the base line brain volume.

### 3.3. ROI-to-ROI analysis

**FIGURE 5:**
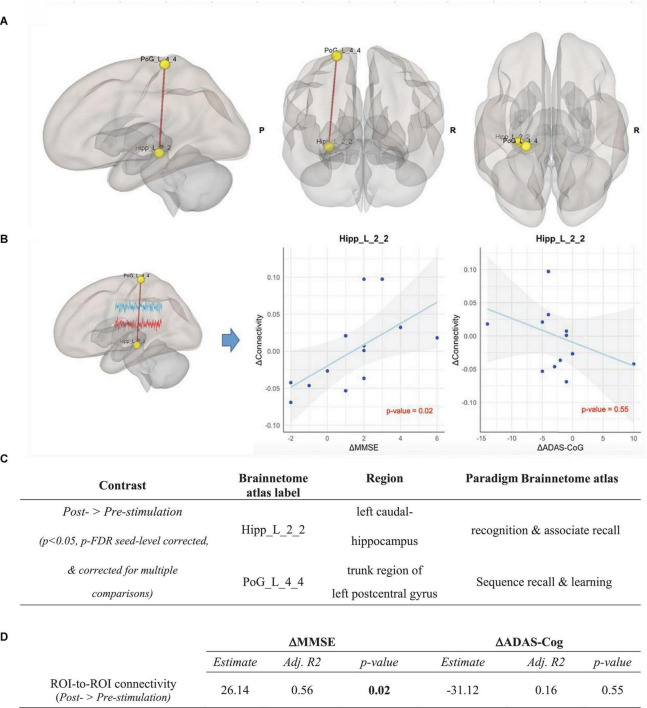
ROI-to-ROI analysis in the intervention group, Contrast: post-stimulation > pre-stimulation. **(A)** Results of ROI-to-ROI analysis in the post- vs. pre-stimulation contrast over all 274 ROIs of Brainnetome Atlas in the intervention group (*p* < 0.05, p-FDR seed-level corrected and corrected for multiple comparisons). Red line indicates increased ROI-to-ROI function al connectivity. **(B)** Illustration of the correlation of the increase of functional connectivity between Hipp_L_2_2 and PoG_L_4_4 with (1) the MMSE scores (left panel) and (2) the ADAS-Cog scores (right panel). Associations are adjusted for age and sex, and *p*-values are Bonferroni-corrected for multiple comparisons. **(C)** Coordinates of ROIs with a significant increase of functional connectivity in the ROI-to-ROI analysis in the intervention group. **(D)** Correlation between the connectivity up-regulation and cognitive measures changes including ΔMMSE and ΔADAS-Cog scores. Associations are adjusted for age and sex, and all *p*-values are Bonferroni-corrected for multiple comparisons. Statistically significant *p*-values are bolded.

Correcting for FD, ROI-to-ROI analysis was performed in both intervention and control groups over all ROIs of the Brainnetome atlas. After correcting for multiple comparisons, we found in the intervention group a significant increase of functional connectivity between the left caudal hippocampus (Brainnetome label: Hipp_L_2_2) and the trunk region of the left postcentral gyrus (Brainnetome label: PoG_L_4_4) from pre- to post-stimulation (*p* < 0.05, p-FDR seed-level corrected and Bonferroni-corrected for multiple comparisons) (see Figure 5). In the control group, no significant ROI-based functional connectivity changes were observed.

To determine the effects of CST in the intervention group compared to the control group, the post- vs. pre-stimulation period contrasts were compared across groups using a repeated measure ANOVA test. The contrast between the changes of the Hipp_L_2_2 ∼ PoG_L_4_4 correlation in the intervention and the control group (ΔConnectivity_*Intervention–Control*_ = ΔConnectivity_*Intervention*_ - ΔConnectivity_*Control*_) showed a significant increase of functional connectivity in the intervention group compared to the control group [*F*(2,23) = 3.9, *p* = 0.029, partial η*^2^* = 0.27]. Importantly, the *post hoc* estimation of our sample size using G*Power 3.1 and IBM SPSS for α = 0.05 showed a power (1-ß) of 0.7.

### 3.4. Seed-to-voxel analysis

**FIGURE 6:**
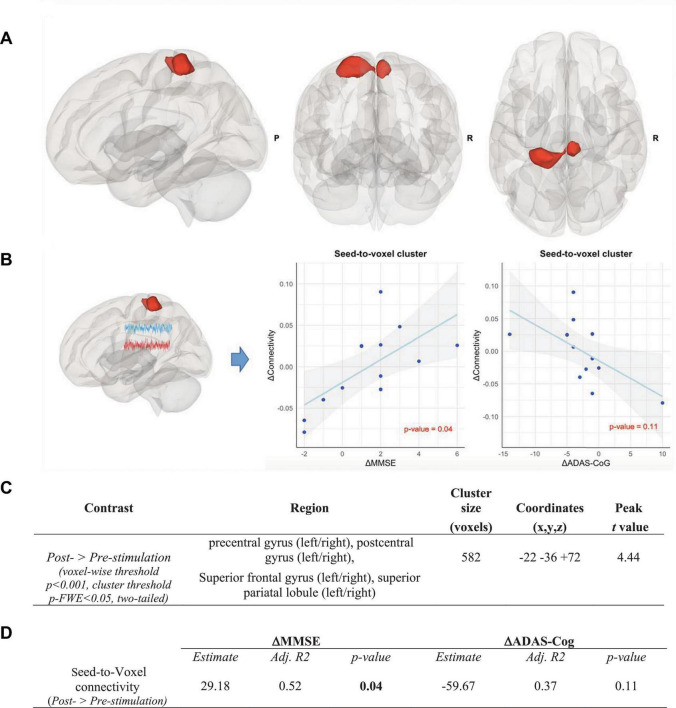
Seed-to-voxel analysis in the intervention group, Contrast: post-stimulation > pre-stimulation. **(A)** Results of seed-to-voxel analysis with the left caudal hippocampus (Brainnetome label: Hipp_L_2_2) as the seed in the post- vs. pre-stimulation contrast in the intervention group (height threshold *p* < 0.001, cluster threshold p-FWE < 0.05, two-tailed). The red color of the cluster indicates the significant increase of functional connectivity. **(B)** Correlation between the increase of functional connectivity in the cluster and (1) the MMSE scores (left panel) and (2) the ADAS-Cog scores (right panel). Associations are adjusted for age and sex, and *p*-values are Bonferroni-corrected for multiple comparisons. **(C)** Coordinates of the clusters with a significant increase of functional connectivity in the seed-to-voxel analysis in the intervention group. **(D)** Correlation between the connectivity up-regulation and cognitive measures changes including ΔMMSE and ΔADAS-Cog scores. Associations are adjusted for age and sex, and all *p*-values are Bonferroni-corrected for multiple comparisons. Statistically significant *p*-values are bolded.

The finding of the ROI-to-ROI analysis was further investigated. A seed-to-voxel analysis using the left caudal hippocampus (Brainnetome label: Hipp_L_2_2) as the seed was performed, which also reflected profound increases in functional connectivity after the CST program. Overlaps of various regions including the left and the right superior frontal gyri, the left and the right superior parietal lobules, the left and the right precentral gyri, and the left and the right postcentral gyri were observed (voxel-wise threshold *p* < 0.001, cluster threshold *p*-FWE < 0.05, two-tailed) (see Figure 6).

### 3.5. Association between connectivity changes and neuropsychological tests

As shown in Figure 5, the increase in connectivity between the left caudal hippocampus (Brainnetome label: Hipp_L_2_2) and the trunk region of the left postcentral gyrus (Brainnetome label: PoG_L_4_4), with memory and learning as classes of testing paradigm, significantly correlated with the improvement of MMSE scores (*p* < 0.05, Bonferroni-corrected for multiple comparison). Expectedly, there was a negative, albeit non-significant, correlation with ADAS-Cog scores among the intervention group participants (see Figure 5). In the seed-to-voxel analysis, the increase in connectivity in the cluster significantly correlated with the improvement of the MMSE score. Although there was a nominally significant negative correlation between the increase in connectivity in the cluster and the ADAS-Cog scores, this correlation did not survive Bonferroni-correction for multiple comparisons (see Figure 6).

## 4. Discussion

In the present study, we investigated the effects of an 8-week CST program in mild to moderate AD patients compared to a no-intervention control group both right after intervention and at a 3-month follow-up. The main parameters of interest were the outcome of cognition, QoL, and associated changes in brain connectivity. We hypothesized that CST compared to the no-intervention control (i) improves the cognition and QoL and (ii) up-regulates cognition-related brain connectivity.

As expected, no significant changes in the neuropsychological assessments within the control group were observed, while the self-rated QoL assessment revealed a significant decrease. In contrast, the analyses within the intervention group showed significant improvements in cognitive performance and neuropsychiatric measures right after the CST period, indicative of positive effects of the CST program. The positive effects of the CST program were further supported by a significant worsening of the majority of the assessments’ measures at the follow-ups, as this renders it improbable that the effects measured right after the CST period are due to repetition of the testing. Remarkably, both groups’ self- and proxy-rated QoL and neuropsychiatric measures were quite homogenous, indicating the reliability of patients’ self-evaluation. These findings are in line with published reviews and meta-analyses reporting that CST effectively improves cognition and QoL in patients with dementia (Buschert et al., 2010; Spector et al., 2012; Aguirre et al., 2013; Chen, 2022; Woods et al., 2012, 2023).

The between-group contrast was significant on the MMSE and self-rated EQ-5D-5L and NPI measures, consistent with the extensive evidence supporting the short-term cognitive benefits for people with mild to moderate dementia participating in CST programs (Woods et al., 2023). Earlier and more recent studies have assessed the effect of cognitive reserve in the outcome neuropsychological approaches in healthy elderly individuals and patients with mild dementia (Wang et al., 2020; Liu et al., 2021). The literature in this regard is fragmented and still inconclusive, nevertheless, previous findings are suggestive of the relevance of cognitive reserve as predictor of the intervention response in CST. In our study, the significant association between the improvement of MMSE and years of education provide more support for the use of cognitive reserve as a predictor of response to CST in mild to moderate AD patients, supporting the argument that patients with higher cognitive reserve are more likely to benefit from CST. However, the lack of association between the baseline total brain volume and the outcomes of our CST may suggest of the independence of the profitability of CST from the brain reserve in patients with mild to moderate AD.

To date, there have been very few studies investigating CST for dementia on structural or functional changes in the brain. In our study, we did not observe any structural changes after CST in the intervention group which is line with a recent report assessing the brain mechanism of CST in patients with dementia (Liu et al., 2021), nevertheless, as structural changes require months or even years to occur, structural analysis in long-term CST maintenance programs is warranted. Next, we explored CST-induced changes in brain connectivity using fMRI. The CST positively affected memory-related left hippocampal connectivity (Brainnetome label: Hipp_L_2_2) with a learning- and memory-associated sub-region of the left postcentral gyrus (Brainnetome label: PoG_L_4_4). The hippocampus is a highly plastic brain region–plasticity allows for the formation of new connections between neurons, a capacity that plays a central role in the development of cognitive ability and high-degree cognitive processes (Wenger and Lövdén, 2016). Environmental enrichment paradigms encompass cognitive stimulation, physical activity, and social interaction which have been shown to induce hippocampal neurogenesis and enhance synaptic plasticity (Lu et al., 2003). A recent study revealed increased resting-state functional connectivity after CST in default mode network (DMN) which supports ongoing cognition (Liu et al., 2021).

The earlier evidence has shown that mental activities in older adults enhance functional connectivity in resting-state brain networks (Chapman et al., 2015). Studies examining the impact of non-pharmacological interventions for dementia on structural and functional brain changes have confirmed that aging brains, with dementia or not, have the capacity for plasticity (Pieramico et al., 2012; Shigihara et al., 2020). It has been proposed that an up-regulated brain connectivity can either reflect neuroplastic modifications of the structural substrate set off by the cognitive training or more flexible use of existing neural pathways through cognitive training, independent of structural changes (Lövdén et al., 2010). Therefore, it appears plausible that the significant up-regulation of connectivity in a neuroplastic region such as the hippocampus was driven by CST. Despite presumed AD-associated hippocampal injury, the hippocampus still retained at least some neuroplasticity to benefit from CST (Rosen et al., 2011).

Furthermore, the seed-to-voxel analysis revealed extended connectivity up-regulation covering partially the left superior parietal lobule, the right and the left precentral gyri, and the left and the right postcentral gyri, previously shown to play a compensatory role in healthy aging and prodromal AD (Behfar et al., 2020). Consistent with our findings of enhanced connectivity in parietal lobes after CST, Liu et al. (2021) reported increased resting-state connectivity in the medial and bilateral parietal cortices following a CST program in patients with mild dementia.

Next, we scrutinized the association between the up-regulation of functional connectivity and the improvement of cognitive performance measures, confirming a significant correlation with MMSE measures. This correlation supports the validity of both imaging and cognitive test results and provides a possible neurobiological underpinning of CST-induced cognitive improvements. Notably, the correlation also proved that the changes in functional connectivity of the hippocampus were consistent with changes in cognitive performance at individual level across subject. The convergence of increased connectivity and improved performance represents an evidence of hippocampal hyperactivation as an attempted compensatory effect (Dickerson and Sperling, 2008). The significantly enhanced connectivity between the hippocampus and parietal regions and its association with improved MMSE measures may support the role of the lateral parietal cortex in episodic memory (Davidson et al., 2008).

Due to the shortage of imaging studies disentangling CST effects on functional connectivity, the mechanisms underlying the CST-induced increase in connectivity remain unclear. Therefore, we here relate our findings to observations made in response to cognitive training as well: in older adults who were cognitively trained, an increase of hippocampal perfusion was seen during memory tasks (van Os et al., 2015). In MCI patients, activation of the hippocampus was steadily observed after memory training, as well as activation of various frontal and parietal cortical regions, not primarily linked to the trained cognitive functions, suggesting that memory training in people with mild brain damage may convey compensatory mechanisms and reallocate cognitive functions to recover the affected functions (Hosseini et al., 2014; van Os et al., 2015). A similar mechanism could apply to CST. CST covers a broader spectrum of cognitive domains than cognitive training. Chapman et al. (2015) suggested another biological pathway that may account for improved brain function in healthy seniors following strategy-based cognitive training. The authors speculated that a cognitive training regime might leave a neural “footprint” on the resting-state signal, such as spontaneous neural activity, which could reflect the aggregation of neurotransmitter-specific receptors in the stimulated areas triggered by strategy-based tasks and enhanced synthesis of vital intra-neuronal molecules required for synaptic functions. Moreover, Valenzuela et al. (2003) showed using MR spectroscopy that the neurochemistry of the medial temporal lobe was modified by prolonged cognitive training in healthy elderly individuals.

The up-regulation of connectivity between the posterior hippocampus and the postcentral gyrus may indicate a dynamic shift from short-range to long-range functional connections: the hippocampi are already functionally disconnected from learning- and memory-associated neighboring structures, such as the entorhinal cortex, in the early stages of AD (DeKosky et al., 2002). In contrast, hippocampal connections to some long-range learning- and memory-associated regions such as the postcentral gyrus remain structurally and functionally relatively intact in the mild to moderate stages of AD (Buckner et al., 2000; Kim et al., 2013). Thus, the CST may have triggered neuronal activity in the hippocampus, and the up-regulated connectivity between the left posterior hippocampus and the trunk region of the left postcentral gyrus may reflect an intact neuroplasticity reserve of the postcentral gyrus, which provides in mild to moderate AD a more fertile field for the sprouting of new connections in a compensatory manner. Previous reports have also suggested that increased benefits observed after implementing cognitive interventions are due to the promotion of complementary neuroplasticity mechanisms (Cespón et al., 2018), by activating and integrating neurons and synapses into pre-existing neural networks (Bamidis et al., 2014).

Considering the multi-domain nature of the interventions, identifying the “active ingredients” of the CST is required for the further improvement of its clinical effectiveness. Herein, the finding of the upregulated resting-state connectivity after CST provides valid insights. The enhanced connectivity in the medial and parietal cortices supports a role of the representation of mental self (Liu et al., 2021). In earlier studies, medial parietal regions were identified as a nodal structure in self-representation (Lou et al., 2004), and it was shown that the lateral parietal cortex may support episodic memory (Davidson et al., 2008). Recent research has revisited the role of self in memory in dementia (Strikwerda-Brown et al., 2019), indicating a reduction in the ability to express episodic memory and future planning due to impairments in self-continuity. In our CST program, cognitive and sensory exercises were built to establish a personal link to the biographies of group members and to elicit conversation about personal experiences and opinions. The cognitive and sensory exercises along with “continuity and consistency between sessions may facilitate and reinstate the sense of self-continuity.” Both autobiographical recall and narrative conditions were found to improve memory in a recent study (Zhang et al., 2020), examining the role of self-referential thinking and memory in amnestic MCI individuals. The authors concluded that the association of information to the “self” in individuals with cognitive impairment provides a useful schema, which relies on the integrity of the autobiographical memory. In this study, our results in exploring the active ingredients of CST are predominantly indicative of alterations in memory domain, which consequently reflects the potentiating role of the relevant tasks in memory domain. These findings in Focusing on these putative “active ingredients” CST may provide a productive refinement in intervention designs.

Our findings must be cautiously interpreted considering some potential limitations. First, the sample size of our study was relatively small, and a larger sample size is required for a confirmation of the results. However, two stimulation sessions per week for 8 weeks in addition to the baseline, post-intervention, and follow-up sessions make this study already quite laborious for a single center. To prove the reliability of the results presented with a significantly larger sample of patients, a multi-center approach is recommendable. Second, the participants could not be blinded. However, this is a common problem of non-pharmacological interventions.

Third, our study design does not disentangle which post-intervention effect is caused by which component of the CST program, or probably even by social aspects of the sessions, to which patients are indirectly exposed.

Fourth, due to the SARS-CoV2-pandemic, data collection encompassing patients at risk for COVID-19 was ethically not justifiable, therefore, data acquisition was stopped, and we used matched imaging and neuropsychological datasets of seven patients from the ADNI database. For these subjects, only MMSE and ADAS-Cog test results were available. Fifth, the umbrella of the environmental enrichment paradigm includes also lifestyle and social habits which can modulate structural and functional brain modifications (Colavitta et al., 2023). We have instructed our participants not to get involved in any new activity during the CST period, nevertheless, compliance to such unmeasured but influential environmental variables by our locally recruited participants and lack of such information from ADNI patients may have confounded our results. Finally, while our study was statistically powered to detect large-size effects, the relatively small sample size may have rendered it underpowered to detect existing smaller effects such as cognitive reserve proxied. Considering the putative effect of the cognitive reserve on the outcomes of CST, we opted to rule out any significant difference in years of education as the most effective proxy for cognitive reserve between the intervention and the control groups.

Major limitations of our study are due to some common challenges in geriatric trials such as recruiting burden, high rate of drop-outs, lack of intervention fidelity and compliance, which may be partially alleviated by an internet-based CST program through digital platforms by overcoming the time-, cost- and place-associated limitations of in-person CST programs. The very large internet-based trials like “Maintain Your Brain (MYB)” (Heffernan et al., 2019), and the multi-center programs like the Finnish Geriatric Intervention Study to Prevent Cognitive Impairment and Disability (FINGER) (Kivipelto et al., 2013) might have the potential to fill this gap by assessing multi-domain interventions in larger cohorts.

## 5. Conclusion

Our results demonstrate the beneficial effects of CST on cognition, QoL, and neuropsychiatric status in mild to moderate AD dementia. Based on the current and previous findings, CST seems to induce short-term global cognitive improvement in earlier stages of AD dementia by activating complementary neuroplasticity mechanisms. Importantly, in light of the small sample, our study attempts to provide imaging-based evidence on the ameliorating effect of CST on cognition. Although further studies in a larger sample, varying age groups, and at multiple centers are warranted, our findings add to the evidence that non-pharmacological therapeutic approaches may be effective in mild to moderate AD dementia.

## Data availability statement

The raw data supporting the conclusions of this article will be made available by the authors, without undue reservation.

## Ethics statement

The studies involving human participants were reviewed and approved by the Ethics Commission of Cologne University’s Faculty of Medicine. The patients/participants provided their written informed consent to participate in this study.

## Author contributions

OAO, QB, EK, A-KF, AC, and GRF designed the experiment. OAO, NR, AC, MK, QB, and RF recruited the study participants. AC and MK performed the cognitive stimulation therapy and collected the neuropsychological data. QB, NR, and OAO collected the imaging data. QB, OAO, GRF, EK, A-KF, SB, RF, and NR conceived the analyses. QB and OAO performed the analyses. QB, NR, MK, AC, SB, A-KF, RF, EK, GRF, and OAO wrote, revised, and approved the manuscript. All authors listed in the author list fully qualify for authorship and contributed significantly to the work.

## Footnotes

1
www.brainmap.org/taxonomy
