# Supplementary material for: Improved connectivity and cognition due to cognitive stimulation in Alzheimer’s disease
Source: Front Aging Neurosci. 2023 Aug 17;15:1140975. doi: 10.3389/fnagi.2023.1140975 (PMC10470843; doi:10.3389/fnagi.2023.1140975)
Supplement: Supplementary file 1 [file Data_Sheet_1.docx]

**Supplementary Materials:**

**Supplementary table 1:** Outcomes of neuropsychological assessments

| Intervention Group | | | | | |
| --- | --- | --- | --- | --- | --- |
| Assessment | Pre-stim. | Post-stim. | Follow-up | pre- vs. Post-stim | Post-stim vs. Follow-up |
|  |  |  |  | *(p-value)* | *(p-value)* |
| MMSE | 19.6±1 | 20.8±1.2 | 18.4±1.3 | **0.02** | **3.4 x 10 ^-3^** |
| ADAS-Cog | 27.1±2.4 | 24.8±3.1 | 26.8±3.1 | **0.04** | 0.06 |
| EQ-5D-5L (self) | 70.3±4.1 | 75.3±4.7 | 71±4.7 | 0.15 | 0.15 |
| EQ-5D-5L (proxy) | 70.3±3.5 | 75±4.3 | 66±4.4 | 0.11 | **0.02** |
| NPI (self) | 10.6±2 | 6.1±1.4 | 11.2±2.1 | **0.01** | **1.6 x 10 ^-3^** |
| NPI (proxy) | 6.1±1.4 | 3.6±1.1 | 6.1±1.7 | **0.03** | 0.05 |
| ADCS-ADL | 60.3±1.7 | 61.8±1.7 | 56.8±2.7 | 0.06 | **0.01** |
|  |  |  |  |  |  |
| Control Group | | | |  | |
| Assessment | 1st test | 2nd test | 1st vs. 2nd test |  | |
|  |  |  |  |  | |
|  |  |  | (*p-value*) |  |  |
| MMSE | 22.8±1.0 | 21.6±1.4 | 0.09 |  |  |
| ADAS-Cog | 20.2±2.3 | 21.8±3.3 | 0.2 |  |  |
| EQ-5D-5L (self) | 85 ± 4.0 | 72.5 ± 4.8 | **0.02** |  |  |
| EQ-5D-5L (proxy) | 68.1 ± 9.4 | 64.4 ± 7.7 | 0.3 |  |  |
| NPI (self) | 7.6±2.2 | 9.9±2.1 | 0.1 |  |  |
| NPI (proxy) | 5.2±2.3 | 5.3±1.7 | 0.5 |  |  |
| ADCS-ADL | 57±4.1 | 57.4±5.4 | 0.4 |  |  |

*Abbreviations: MMSE: Mini-Mental State Examination; ADAS-Cog: the Alzheimer’s Disease Assessment Scale, cognitive subsection; EQ-5D-5L: the European Quality of Life Five Dimension with Five Levels; NPI: The Neuropsychiatric Inventory; ADCS-ADL: the Alzheimer’s Disease Cooperative Study-Activities of Daily Living Inventory*

*Statistically significant p-values are bolded.*

**Supplementary Figure 1**: Association between the total brain volume and significant outcomes of CST

**
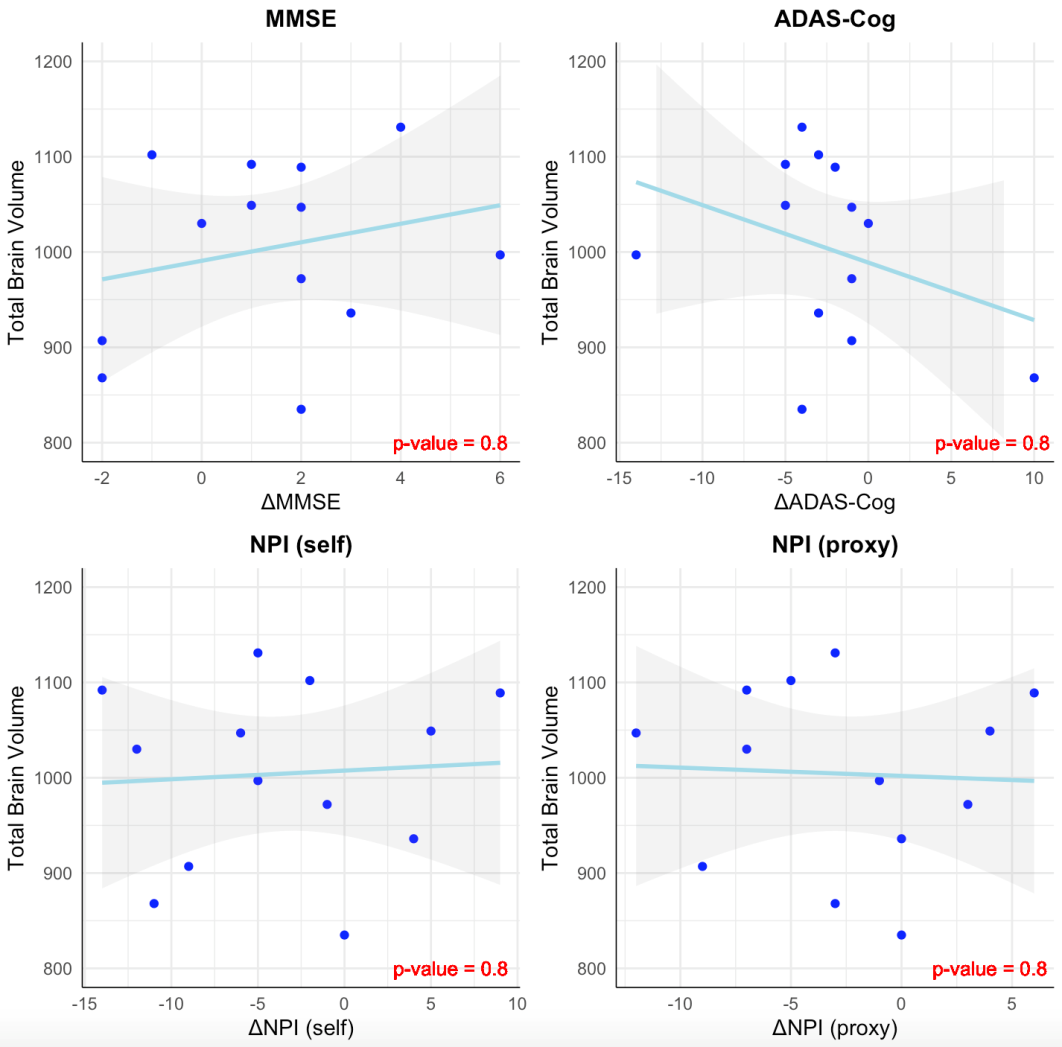
**

| Total Brain Volume | **ΔMMSE** | | | **ΔADAS-Cog** | | |
| --- | --- | --- | --- | --- | --- | --- |
|  | *Estimate* | *Adj. R2* | *p-value* | *Estimate* | *Adj. R2* | *p-value* |
|  | 5 x 10 ^-3^ | -0.03 | 0.8 | -0.01 | 0.02 | 0.8 |
|  | **ΔNPI (self)** | | | **ΔNPI (proxy)** | | |
|  | *Estimate* | *Adj. R2* | *p-value* | *Estimate* | *Adj. R2* | *p-value* |
|  | 4 x 10 ^-3^ | -0.08 | 0.8 | -2 x 10 ^-3^ | -0.08 | 0.8 |

*Correlation between the total brain volume as an indicator of brain reserve and post- vs. pre-stimulation changes of MMSE, ADAS-Cog, NPI (self) and NPI (proxy) scores in the intervention group. The post- vs. pre-stimulation changes are respectively represented by ΔMMSE, ΔADAS-Cog, ΔNPI (self) and ΔNPI (proxy). All p-values are Bonferroni-corrected for multiple comparisons.*
